# *Pantoea* abscess mimicking sarcoma in a HTLV‐1‐infected Indigenous Australian man: Case report and literature review

**DOI:** 10.1002/ccr3.7351

**Published:** 2023-05-18

**Authors:** Maja Susanto, Jacki Dunning, Rusheng Chew

**Affiliations:** ^1^ Infectious Diseases Unit Redcliffe Hospital Redcliffe Queensland Australia; ^2^ Department of Surgery Alice Springs Hospital Alice Springs Northern Territory Australia; ^3^ Infectious Diseases Unit Alice Springs Hospital Alice Springs Northern Territory Australia; ^4^ Faculty of Medicine University of Queensland Brisbane Queensland Australia; ^5^ Centre for Tropical Medicine and Global Health University of Oxford Oxford UK

**Keywords:** abscess, Enterobacteriaceae, HTLV‐1, Indigenous Australians, *Pantoea*, sarcoma

## Abstract

Gram‐negative bacteria of the genus *Pantoea* are emerging bacterial causes of diverse sporadic and outbreak‐linked infections. Chronic *Pantoea* abscesses are unusual and may give rise to a differential diagnosis of malignancy. Foreign body retention and host immune defects may be risk factors for such chronic infections.

## Graphical abstract

This is the second reported case of *Pantoea* pseudotumor in the literature, and the first in a HTLV‐1‐infected patient. At the time of writing, the patient remained systemically well despite persistence of the large lesion (demarcated by the arrows on this image of a contrast CT sagittal section of the right leg) for 8 years in the absence of surgical treatment. The factors predisposing to the establishment of chronic *Pantoea* soft tissue infection remain unclear, and may be related to both host and pathogen characteristics.

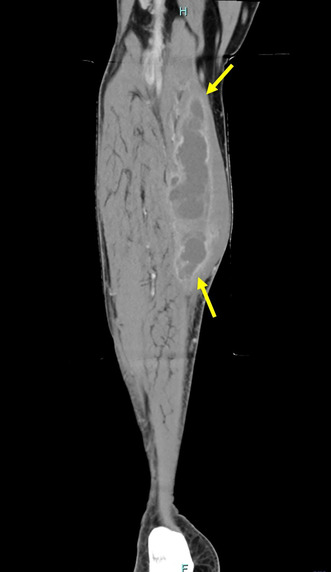

## 1 INTRODUCTION

Members of the genus *Pantoea* are Gram‐negative motile non‐capsulated and non‐spore forming rods belonging to the Enterobacteriaceae family. The genus was established in 1989 and its 20 species are ubiquitous, being found in plants, insects, and animals as well as water, soil, and air.^1^ *Pantoea* has traditionally been regarded as plant pathogen, but certain species are increasingly recognized as emerging opportunistic causes of human disease, including nosocomial outbreaks.^2^, ^3^ As such, while immunocompromise and contact with plant material and healthcare environments are risk factors for infections caused by *Pantoea*, its widespread ecological niche implies that sporadic infections without these risk factors are likely to occur.^1^, ^3^

## 2 CASE REPORT

A 55‐year‐old Indigenous Australian male living in remote Central Australia was admitted to Alice Springs Hospital in December 2015 for pain and swelling at the right calf resulting in a limp. For context, Central Australia is a rural region of over 1,000,000 km^2^ comprising approximately 10% of the total Australian landmass. It is sparsely populated by about 60,000 people.^4^ Of these, approximately 40% are Indigenous Australians who are more likely to suffer from numerous chronic diseases and have poorer health literacy than their non‐Indigenous counterparts.^5^ Alice Springs Hospital is the referral facility for this vast remote area.

**FIGURE 1:**
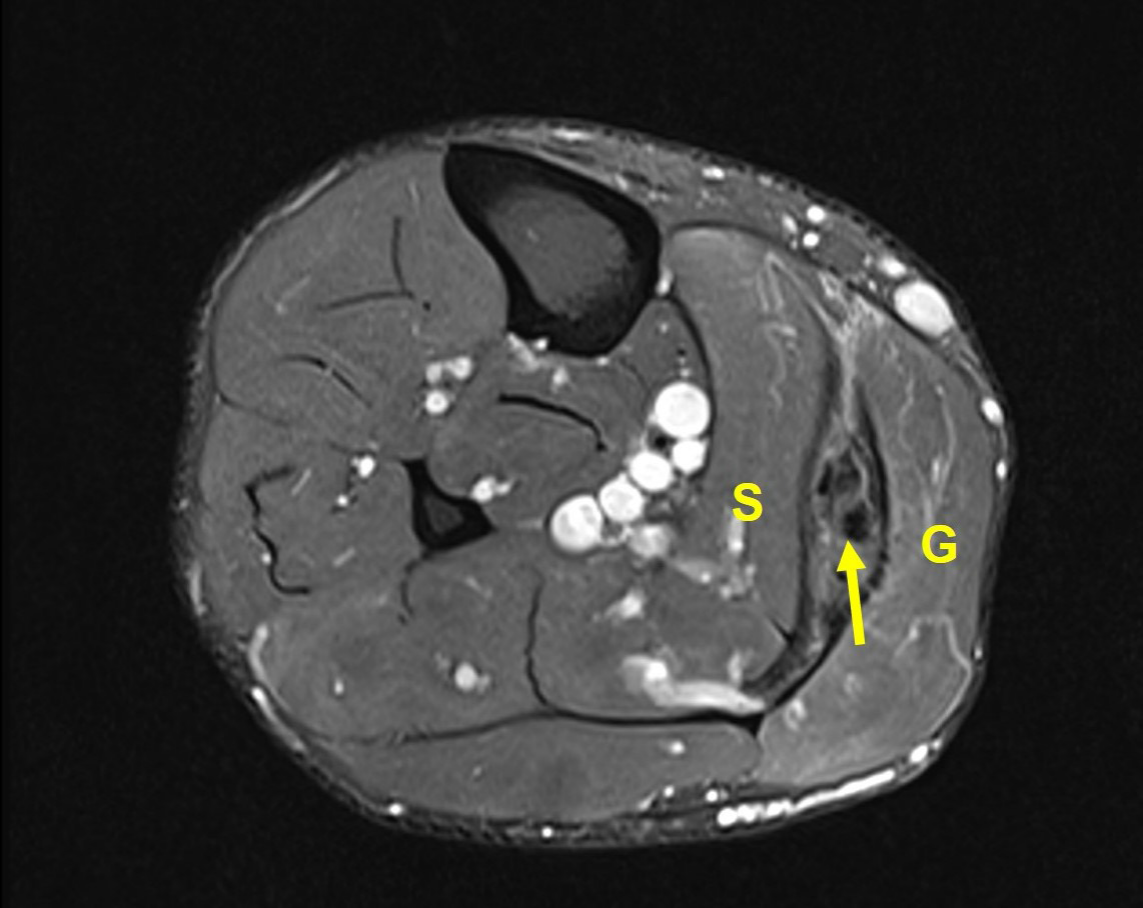
MRI axial section of the right calf showing the location of the pseudotumor (arrow) between the gastrocnemius (G) and soleus (S) muscles.

The patient's relapsing–remitting symptoms had begun in 2012 following a fall which, according to him, did not cause traumatic injury or skin penetration. He was reviewed in the orthopedic clinic in 2014 where he was noted to have an antalgic gait with a range of motion at the right knee of 90–140° and crepitus on movement, and a palpable medial gastrocnemius lesion. MRI of the right leg showed heterogeneous thickening of the medial gastrocnemius/soleus measuring 35 × 28 × 147 mm with unusual tubular extension into the central aspect of the medial gastrocnemius favoring post‐injury haematoma, but raising the possibility of a neoplastic process (Figure 1). Unfortunately, he was lost to hospital follow‐up until October 2015 when a further orthopedic outpatient review showed no change in the morphology or character of the lesion and continued observation was recommended.

His past medical history included poorly controlled type 2 diabetes mellitus complicated by macroalbuminuria, hypertension, hyperlipidemia, obesity, complete heart block requiring pacemaker insertion, osteoarthritis of the right knee and left shoulder, and right leg varicose veins. His regular medications were aspirin 100 mg daily, atorvastatin 40 mg daily, gliclazide MR 120 mg daily, metformin XR 2 g daily, perindopril/indapamide 10/2.5 mg daily, sitagliptin 100 mg daily, and tadalafil 20 mg as required. On examination at the time of admission, there were distended superficial veins below the knee, and marked hard swelling and tenderness of the ankle and right calf to the level of the popliteal fossa. He was afebrile with a pulse rate of 96, respiratory rate of 22, blood pressure of 99/90 mmHg, and oxygen saturation of 97% breathing air on admission. C‐reactive protein was raised at 177 mg/L (normal range 0–5), and he had a neutrophil leukocytosis with total white cell and neutrophil counts of 16.3 × 10^9^/L (4.0–11.0) and 10.4 × 10^9^/L (2.0–7.5), respectively. Blood cultures were not performed.

**FIGURE 2:**
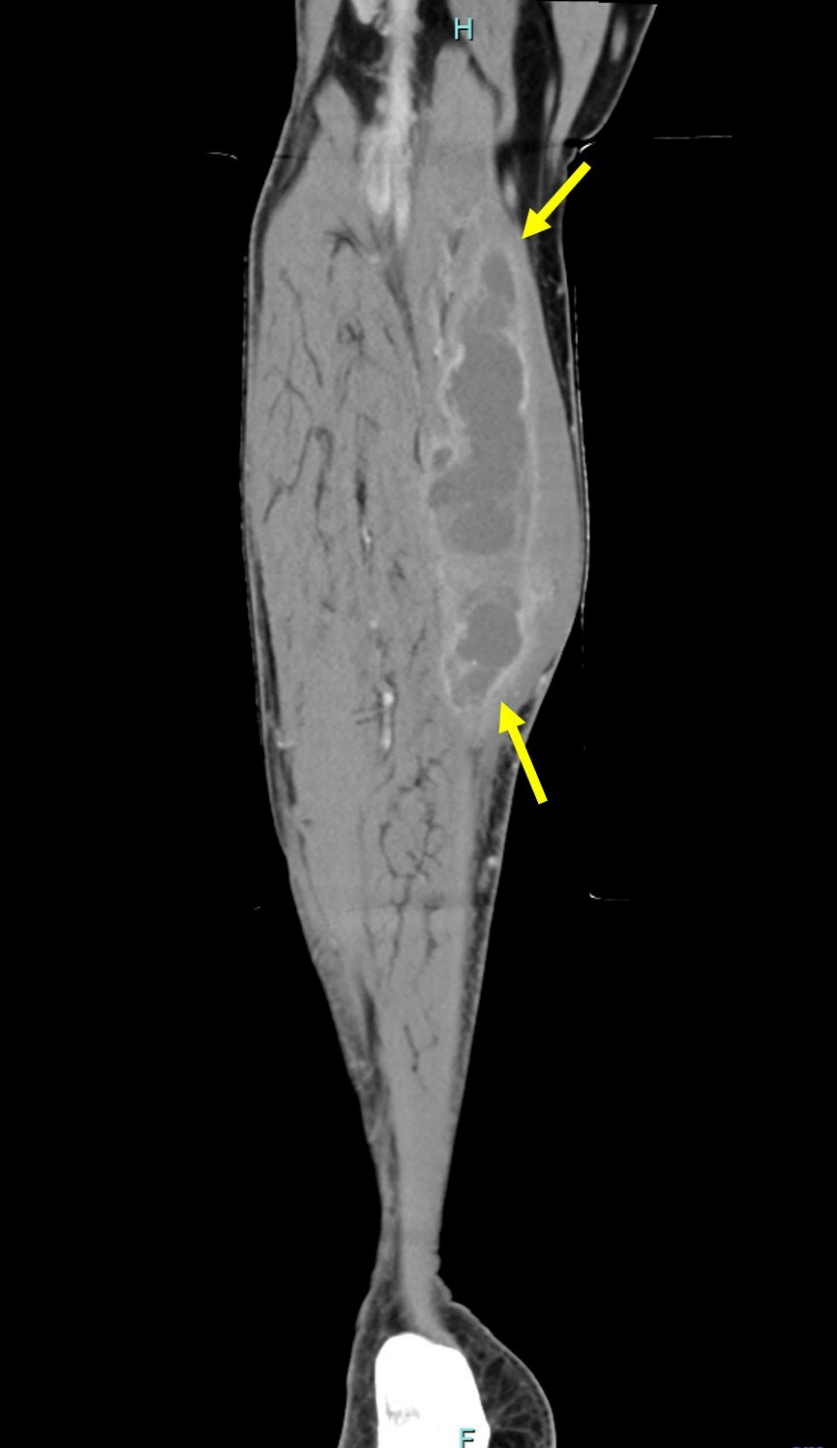
Contrast CT sagittal section of the right leg showing the extent of the multi‐loculated pseudotumor (arrows) within the calf.

A CT scan with contrast was performed to characterize the lesion further. This again showed a cystic morphologically heterogenous structure, but also that it had increased in size to 38 × 49 × 156 mm with a larger solid component, again raising the possibility of sarcoma (Figure 2). This differential diagnosis was also supported by the peripherally enhancing, multi‐loculated nature of the lesion. The patient remained an inpatient under the General Surgical team for 3 days, where he received empirical cefazolin 1 g eight‐hourly. Aspiration of the lesion for histological and microbiological examination was planned, but deferred by the patient to February 2016 when it was performed under ultrasound guidance.

**FIGURE 3:**
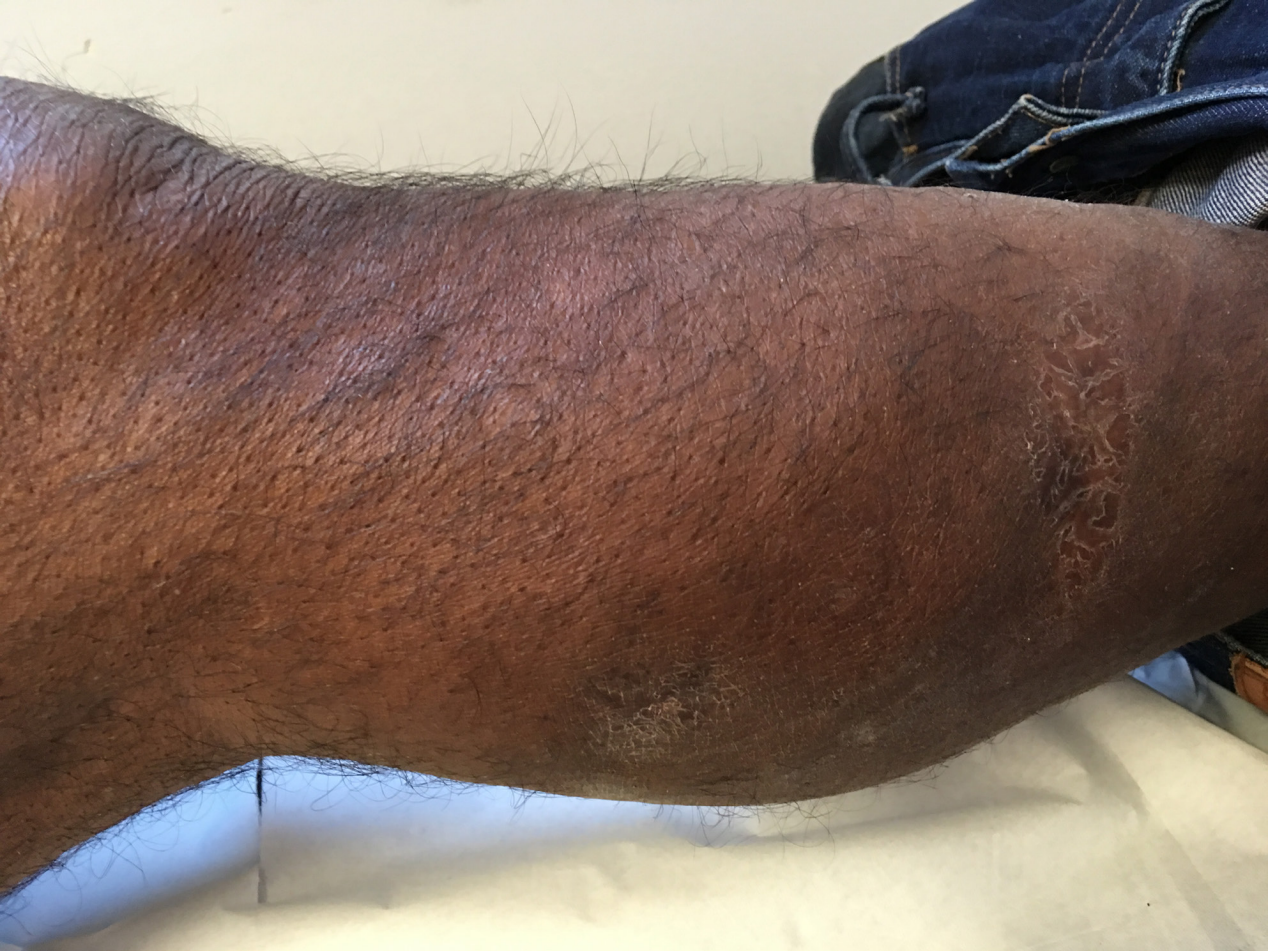
Photograph of the right calf mass 4 years after its appearance.

The aspirate demonstrated an inflammatory exudate comprising primarily neutrophils and pigment‐laden macrophages compatible with an abscess. No malignant cells were seen, but culture yielded pure growth of *Pantoea* spp. susceptible to amoxicillin/clavulanate, cefazolin, ciprofloxacin, gentamicin, and trimethoprim/sulfamethoxazole but resistant to ampicillin. Identification to species level was precluded by laboratory technological limitations but, given the culture result, the patient was referred to the Infectious Diseases clinic where he was seen in April 2016. He reported never feeling systemically unwell or febrile, and that, subsequent to the aspiration, the pain and swelling had improved to the extent he was able to walk normally. The calf mass remained palpable and hard to touch (Figure 3), but CRP had decreased to 11 and he no longer had a leukocytosis. Investigations for immunodeficiency revealed no lymphocyte deficiency and negative serology for HIV and *Strongyloides*, but he was infected with HTLV‐1, in keeping with the very high prevalence in Indigenous Central Australians.^6^ A detailed occupational and exposure history was taken, in which he denied performing agricultural work or gardening in the course of his work as an Aboriginal Health Worker or recreationally, but for cultural reasons would go into the desert surrounding his rural community when required.

To confirm that the lesion was, indeed, an abscess a second aspiration and tissue core biopsy was performed. Again, no malignant cells were seen and pure growth of *Pantoea* spp. was cultured. Insufficient tissue was obtained to draw a definitive histological conclusion, but the patient refused a second attempt as well as any surgical intervention. As such, he was commenced on amoxicillin/clavulanate 875/125 mg in May 2016 for 6 weeks, ciprofloxacin being contraindicated because of his history of arrhythmia.

Despite successful completion of antibiotic therapy, the lesion was still obvious at follow‐up in October 2016 and the patient reported similar symptom exacerbations in the preceding months which would spontaneously resolve. His case was, thus, discussed at a specialist sarcoma multi‐disciplinary meeting where the consensus opinion was that an abscess, rather than malignancy, was the most likely diagnosis. Ongoing surveillance with imaging was recommended but the patient was lost to follow‐up until 2020, when an ultrasound scan requested by his general practitioner showed persistence of the mass which had organized into three separate collections measuring 150 × 25 mm, 27 × 10 mm, and 31 × 28 mm. The patient declined further intervention for this problem and, at the time this report was written in 2022, remained systemically well. Aside from the 6 weeks of amoxicillin/clavulanate commenced in 2016, he had not had any extended antibiotic courses for the abscess.

## 3 DISCUSSION

**FIGURE 4:**
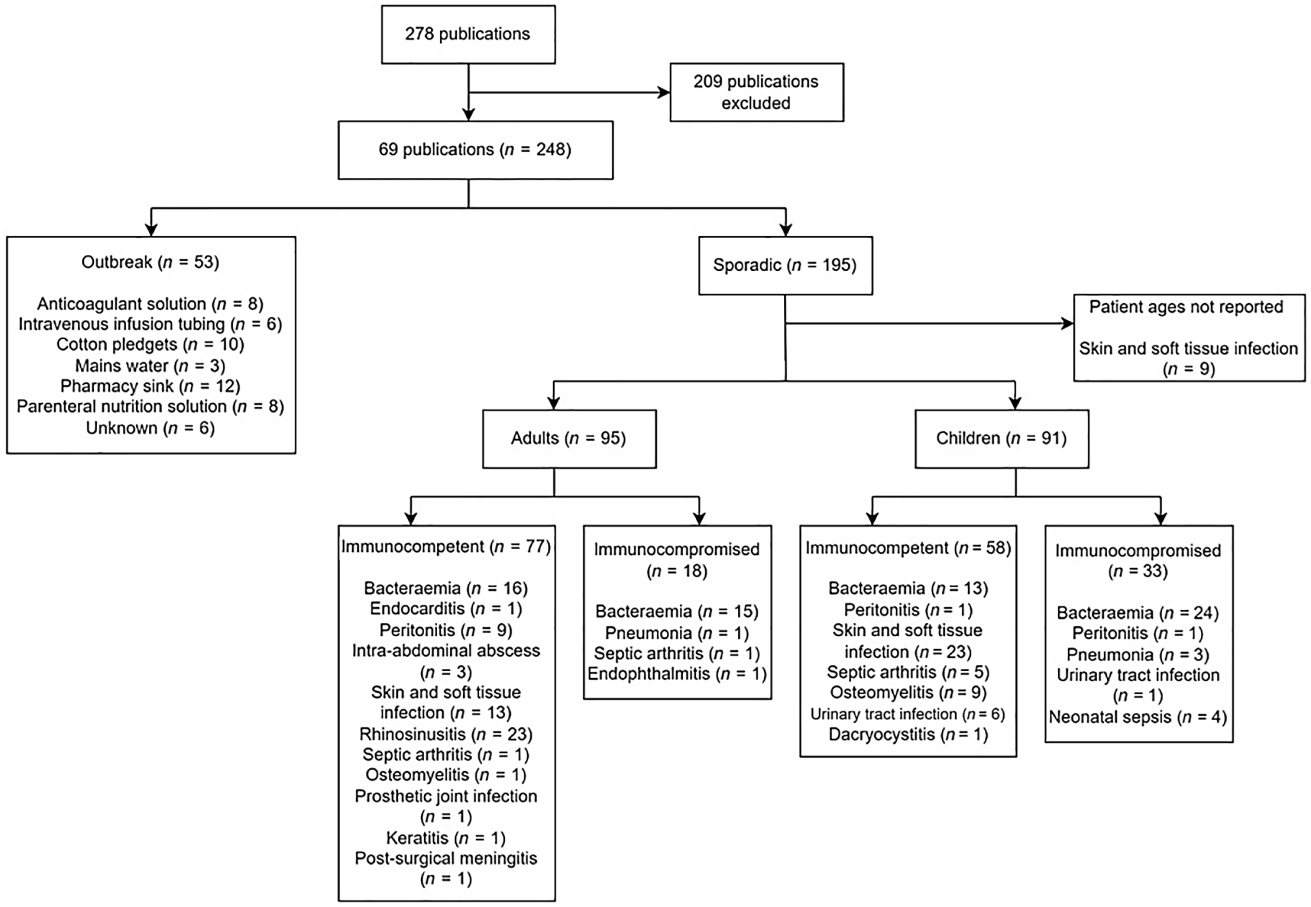
Literature review flowchart showing sources and clinical syndromes for outbreaks and sporadic *Pantoea* infections, respectively. Immunocompromise in the pediatric population included age <28 days.

To place our case in context, we searched the MEDLINE database on September 27, 2022 to determine the spectrum of pathologies caused by *Pantoea* and associated risk factors. We used the search term ‘*Pantoea*’ with no restriction on publication date, and applying the “human” and “English language” filters. This search yielded 278 results, from which 69 case reports, case series, and outbreak reports were selected for data extraction. Patients included in these publications were stratified by immune status and age, with children defined as those <18 years old. We also extracted data on clinical syndromes of sporadic cases, as well as sources of outbreaks. The literature review is summarized in the flowchart shown in Figure 4.

As is evident from the flowchart, *Pantoea* infections are rare, with only 248 cases reported worldwide between 1991 and 2022, 53 of which were outbreak cases stemming from various contaminated environmental sources.^7^, ^8^, ^9^, ^10^, ^11^, ^12^, ^13^ Sporadic adult and pediatric cases were seen in equal proportions, with immunocompetent patients greatly outnumbering their immunocompromised counterparts. Bacteraemia,^14^, ^15^, ^16^, ^17^, ^18^, ^19^, ^20^, ^21^, ^22^, ^23^, ^24^, ^25^, ^26^, ^27^, ^28^, ^29^, ^30^, ^31^, ^32^, ^33^, ^34^, ^35^, ^36^ followed by skin and soft tissue infections (SSTI),^31^, ^32^, ^37^, ^38^, ^39^, ^40^, ^41^, ^42^ were the commonest manifestations of *Pantoea* infection, but the range of syndromes was wide, including peritonitis,^32^, ^43^, ^44^, ^45^, ^46^, ^47^, ^48^, ^49^, ^50^, ^51^, ^52^ bone and joint infection,^32^, ^53^, ^54^, ^55^, ^56^, ^57^, ^58^, ^59^, ^60^ intra‐abdominal abscess,^61^, ^62^, ^63^ pneumonia,^31^, ^64^ urinary tract infection,^31^, ^32^ ocular infection,^65^, ^66^, ^67^, ^68^, ^69^, ^70^, ^71^ and rhinosinusitis.^72^ One case each of endocarditis,^15^ prosthetic joint infection,^55^ and post‐neurosurgical meningitis^73^ was also reported, as were four cases of neonatal sepsis.^74^, ^75^ Only in 19 patients, all of whom had either SSTI,^37^, ^41^, ^42^ septic arthritis,^53^, ^56^, ^58^, ^59^ or ocular infection^66^, ^69^ was there a history of penetrating plant trauma, indicating that this risk factor is less important than previously assumed. The vast majority (84%) of infections were caused by *P. agglomerans*, with only four other culprit species reported: *P. dispersa*,^16^, ^17^, ^25^, ^27^, ^28^, ^72^ *P. ananatis*,^19^, ^21^, ^68^ *P. calida*,^24^, ^73^ and *P. stewartii*.^18^ In four other cases, identification to species level was unable to be performed,^39^, ^66^, ^67^, ^71^ like in ours. However, the diversity of *Pantoea* species is not adequately differentiated by many laboratory identification methods and, as such, many cases attributed to *P. agglomerans* may, in fact, have been caused by other species.^76^, ^77^, ^78^

Our patient, therefore, exhibited a very atypical manifestation of a *Pantoea* SSTI mimicking malignancy, to the extent that both clinical and radiological findings raised concerns for sarcoma. Sarcoma is highly unlikely in our case, given the repeated pure growth of *Pantoea* spp., the long intervening period without death or deterioration, and the expert opinion provided by the specialist sarcoma multi‐disciplinary meeting. While he denied any penetrating trauma, whether plant‐related or otherwise, it is possible that micro‐abrasions may have occurred when he fell, providing a portal of entry. Furthermore, given his rural residence, it is likely that plant or animal material was present on the ground, and his poor diabetic control likely contributed to the development and progression of infection.

Only one other case of *Pantoea* pseudotumor has been reported, although this patient from India provided a clear history of penetrating plant trauma due to his work in agriculture.^42^ Like our case, this patient also reported a distant history of a fall 4 years prior to presentation. Regardless, both cases, despite the different management approaches taken, resulted in good outcomes. While surgical drainage of the lesion is ideal, as in the latter case, it is interesting that in our patient the infection was successfully contained with him remaining well 10 years after symptom onset, even without surgical management. An important clinical lesson, therefore, is that foreign bodies, especially of plant origin which are not well‐visualized on plain radiographs, may be retained following such trauma and act as foci of chronic inflammation leading to pseudotumor formation.

Our case raises some interesting questions. The first is whether the patient's HTLV‐1 infection further predisposes to the establishment of a chronic bacterial infection. HTLV‐1, unlike HIV, does not result in overt immunodeficiency, but associations between HTLV‐1 and non‐bacterial infections, such as scabies and strongylodiasis, are well‐described.^79^ However, little is known about how HTLV‐1 mediates concurrent bacterial infections,^80^ making this an important research question of clinical significance for the many infected Indigenous Central Australians in whom rates of bacterial infection far exceed those of their non‐Indigenous countrymen,^79^ as well as people living with this neglected tropical disease worldwide.

The second question arises from the ability of *Pantoea* to secrete products with bioremediative and immunogenic potential, facilitating its adaptation to diverse ecological niches, including in hostile environments.^1^ It may well be that this has aided the establishment of a well‐contained infective focus in our patient, given that macrophage activation and epithelial‐mesenchymal transformation due to inflammatory mediators released by *P. agglomerans* leading to fibrosis has been recently reported.^81^ Such a process may have succeeded in walling off the abscess, thus preventing cell‐mediated immunity from eradicating the infection but also preventing the development of sepsis. Unfortunately, our laboratory was unable to speciate the causative organism, but other species may also have this capability. As such, research to elucidate the mechanisms of action of pathogen mediators released during *Pantoea* infections may be clinically useful.

## 4 CONCLUSION

In conclusion, we have reported, to our knowledge, the second case of *Pantoea* pseudotumor in the literature, and the first in a patient with HTLV‐1 infection. Through our analysis of this case, we have also identified areas in which further research will have clinically beneficial implications.

## CONSENT

The authors confirm that the patient provided written consent for publication of this report.

## AUTHOR CONTRIBUTIONS

**Maja Susanto:** Formal analysis; investigation; writing – original draft. **Jacki Dunning:** Formal analysis; investigation; writing – original draft. **Rusheng Chew:** Conceptualization; formal analysis; investigation; methodology; supervision; writing – original draft; writing – review and editing.

## FUNDING INFORMATION

None required.

## CONFLICT OF INTEREST STATEMENT

The authors declare no conflicts of interest.

## Data Availability

Data sharing not applicable to this article as no datasets were generated or analysed during the current study.
